# *In vivo* evaluation of hypolipidaemic and antioxidant effects of aqueous extract from Caco seeds (*Chrysobalanus icaco* L.) and action mechanisms by *in silico* prediction in a model of hypertriglyceridaemia

**DOI:** 10.1017/S0007114526106667

**Published:** 2026-06-28

**Authors:** Abel Arce-Ortiz, Carlos Alberto Lobato-Tapia, Sergio Esteban Moreno-Vázquez, Cristian Jiménez-Martínez, Zendy Evelyn Olivo-Vidal, Luis Jorge Corzo-Ríos, Luis Fernando García-Melo, Xariss M. Sánchez-Chino, Gabriel Alfonso Gutiérrez-Rebolledo

**Affiliations:** 1 Departamento de Salud, El Colegio de la Frontera Sur - Unidad Villahermosa, Mexico; 2 Ingeniería en Biotecnología, Universidad Politécnica Metropolitana de Puebla, Mexico; 3 Instituto Politécnico Nacional Escuela Nacional de Ciencias Biologicas, Mexico; 4 Instituto Politecnico Nacional Unidad Profesional Interdisciplinaria de Biotecno, Mexico; 5 Departamento de Química, Universidad Autonoma Metropolitana, Mexico; 6 Secretaria de Ciencia, Humanidades, Tecnología e Innovación, Mexico

**Keywords:** *Chrysobalanus icaco*, Seeds, Flavonoids, Oxidative stress, Hypertriglyceridaemia, Molecular docking

## Abstract

*Chrysobalanus icaco* L. (Caco) is a fruit tree distributed in tropical areas of Africa and America. Its seeds are a rich source of bioactive compounds, and their consumption could have a positive impact on human health during dyslipidaemias. The objective of this study was to evaluate the hypolipidaemic and antioxidant activities of aqueous extract of Caco seeds in an *in vivo* model of hypertriglyceridaemia induced by Triton WR-1339 (tyloxapol). Phytochemical characterisation revealed saponin and phytic acid contents of 4730 ± 190 µg of saponin equivalents and 1·0 ± 0·05 µg phytic acid equivalents g^–1^ of sample, respectively. Phenolic acids and flavonoids (ellagic acid, apigenin-7-O-glucuronide and myricetin, among others) were identified by HPLC-quadrupole time-of-flight (TOF) -MS. Aqueous extract of Caco seeds was administered once daily for three consecutive days at two doses (150 and 600 mg/kg) in male CD1 mice, where treatment with 600 mg/kg reduced serum TAG levels by 64 % compared with control, decreased oxidative damage to lipids and proteins and modulated superoxide dismutase and glutathione peroxidase activity in hepatic tissue. Complementary *in silico* molecular docking analyses suggested a potential interaction of apigenin-7-O-glucuronide with lipid metabolism-related enzymes. These findings indicate that *C. icaco* L. seeds may be considered a promising source of bioactive molecules for the treatment and management of early phases of dyslipidaemias, as evidenced in an acute model, but their full potential in chronic stages merits further research.

## Graphical abstract

**Figure:**
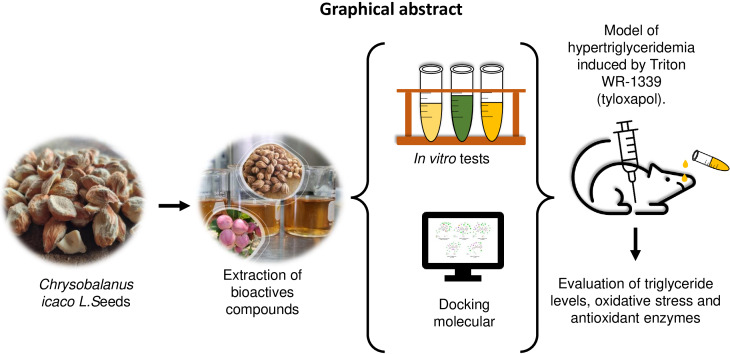

Hyperlipidaemia is a metabolic disorder characterised by increased concentrations of total cholesterol, TAG and LDL-cholesterol, which are major risk factors for the development of CVD: atherosclerosis, hypertension, CHD, diabetes and obesity^(1–3)^. Among these, hypertriglyceridaemia induces hepatic dysfunction due to excessive TAG accumulation and enhanced synthesis of VLDL-cholesterol^(4)^. This overload promotes mitochondrial imbalance and reactive oxygen species generation, leading to oxidative stress (OS), which contributes to chronic degenerative diseases^(4)^. OS arises when the equilibrium between endogenous antioxidants and reactive molecules such as reactive oxygen species and reactive nitrogen species is disrupted, causing cellular and systemic damage^(5–7)^.

To counteract oxidative damage, organisms rely on enzymatic antioxidants such as superoxide dismutase (SOD), glutathione peroxidase (GPx) and catalase, as well as non-enzymatic antioxidants such as glutathione^(8)^; however, these defensive systems are often overwhelmed during early and late phases of dyslipidaemia, making exogenous sources of antioxidants necessary.

Plant-derived foods are particularly relevant, as they contain secondary metabolites or bioactive compounds (BC) (phenolic compounds, saponins, terpenoids, alkaloids, phytates, phytosterols and vitamins) that exert regulatory effects in human metabolism and physiology. Beyond their antioxidant activity, these compounds may also regulate serum TAG and total cholesterol levels and display anti-inflammatory and antioxidant properties^(9–12)^.

Caco (*Chrysobalanus icaco* L.) is a fruit tree distributed in tropical areas of Africa and North and South America. In Mexico, it is found in Chiapas, Yucatan, Oaxaca, Quintana Roo, Guerrero and Tabasco^(13)^. It is considered a multipurpose plant: ripe fruits are used in preserves, jellies and beverages^(14)^, while leaves, roots, bark and seeds are used in herbal medicine for malaria, haemorrhages, gastrointestinal disorders, inflammation and metabolic syndrome regulation, among others^(15)^.

Previous studies have shown that ethanolic extract of Caco seeds, administered orally (200 mg/kg per d) for 28 d in rats fed a fat-rich diet, significantly reduced total cholesterol, TAG and VLDL-cholesterol, while increasing high-density lipoproteins HDL-cholesterol compared with healthy controls^(16)^. These findings confirm the hypolipidaemic effect of repeated administration in healthy animals; however, the impact of Caco seed extracts in acute dyslipidaemia, such as hypertriglyceridaemia, and their role in hepatic OS remain unexplored.

Aqueous extract of Caco seeds (AECS) has been reported to contain polyphenols, acylsugars, lignans, xanthones and flavodoxins^(9)^. These compounds confer antioxidant capacity by maintaining or restoring redox balance, thereby preventing the onset and exacerbation of OS during pathological conditions^(10,12,15)^. Moreover, AECS has shown a favourable safety profile. Arce-Ortiz *et al.* ^(9)^ reported a median lethal dose > 2 g/kg in CD1 mice after a single intragastric administration, suggesting low acute toxicity.

Given this background, the tyloxapol-induced hypertriglyceridaemia model provides a suitable acute setting to evaluate whether AECS can attenuate lipid dysregulation and oxidative damage in hepatic tissue. The present study therefore aimed to identify and quantify the phytochemical compounds of AECS, characterise their profile by HPLC-quadrupole time-of-flight-MS (HPLC-QTOF-MS) and evaluate their hypolipidaemic and antioxidant effects *in vivo* in male CD1 mice, complemented by *in silico* docking analysis to propose a possible hypolipidaemic mechanism.

## Methodology

### Biological material and conditioning of Caco seeds

Mature Caco fruits were collected at Bahía de Paredón, in the municipality of Tonalá, Chiapas, Mexico (16°03′03″N, 93°52′00″W)^(9)^. To obtain the seeds, the fruits were washed and disinfected, and the white cotton-like pulp and woody bark were removed. The seeds were dried in a convection oven at 40°C for 72 h (ECOSHEL 9053A) and then ground (Krups mill) and sieved through a no. 14 mesh to achieve a uniform particle size of ≤ 1·41 mm. The ground seeds were defatted with hexane for 6 h using the Soxhlet method (1:10) and then placed in trays at room temperature for 12 h to eliminate the excess solvent^(9)^. Finally, the solvent-free dry sample (flour) was stored in polyethylene bags and kept refrigerated until use.

### Extract preparation

The extract was prepared as described by Arce-Ortiz *et al.* ^(9)^. For this purpose, 100 mg of defatted flour was weighed and added to 10 ml of distilled water, obtaining a concentration of 10 mg/ml. The mixture was then placed in an ultrasonic bath (Ultrasonic Cleaner, 010S, CGOLDENWALL) at 42 kHz for 30 min and then passed through a nylon filter of pore size 22 µm, poured into amber-coloured vials and stored at 4°C until use.

### Determination of the phytochemical contents of Caco seed extracts

A microplate reader (Multiskan Go Thermo Scientific) was used in these determinations to allow adjustment to the micromethod and different wavelengths, as described below.

The methodology described by León-Roque *et al.* ^(17)^, adapted to microplates, was used to determine the saponin content. Samples were analysed in triplicate and read at 554 nm. A diosgenin standard curve was used to calculate the saponin content. The results were expressed as mg of diosgenin equivalent (DGE)/g sample (mg DGE/g sample).

Phytate concentration was determined according to the methodology described by Yathish *et al.* ^(18)^, with modifications. Samples were analysed in triplicate and read at 490 nm. A phytate acid standard curve was used to calculate the phytate content. The results were expressed as mg of phytate acid equivalent (PAE)/g sample (mg PAE/g sample).

Trypsin inhibitor (TI) activity was determined according to the methodology described by Senanayake *et al.* ^(19)^, with modifications. The results were expressed as mg of pure trypsin inhibited/g sample (trypsin inhibitr activity (TIA)/g sample).

### Metabolomic profiling by HPLC coupled to quadrupole time-of-flight MS

Total phenolic compounds were identified by HPLC-QTOF-MS, adapting the method of Du *et al.* ^(20)^. The process was performed on an HPLC (Agilent 1260, Agilent Technologies) equipped with an Agilent 6520 Accurate-Mass QTOF LC-MS/MS (Agilent Technologies). Before being transferred to HPLC vials, the sample was centrifuged and filtered with a 0·22 μm PTFE syringe filter. A Luna C_18_ column (5 μm 250 × 4·6 mm, Phenomenex) was used. The mobile phases included two solvents: A (water + 0·1 % formic acid) and B (acetonitrile + 0·1 % formic acid). The following gradient conditions were used: 5–15 % (0–40 min), 60 % B (40–65 min), 95 % B (65–75 min), 95 % B (75–80 min) and 5 % B (80·1–100 min). The column temperature was maintained at 40°C, the injection volume was 50 µL and the flow rate was adjusted to 0·7 ml/min. MS/MS peak identification was carried out in both negative and positive modes, and mass spectra were obtained within the range of *m/z* 50–500 atomic mass units (amu) and a fragmenter voltage of 175 Volts (V). The nitrogen gas temperature was set at 300°C, and the flow rate was set at 5 L/min. The enveloping gas temperature was set at 250°C, and the flow rate was set at 11 L/min, with an atomiser gas pressure of 45 psi. Data collection and analysis were carried out using the qualitative software Agilent LC-ESI-QTOF-MS/MS Mass Hunter B.03·01 (Agilent Technologies).

### Evaluation of in vivo hypolipidaemic and antioxidant activities

#### Biotherium conditions and characteristics of the experimental animal species

Male CD1 mice of 25 ± 5 g were used for the induction of hypertriglyceridaemia. These animals were sourced from the biotherium PROPECUA S.A. de C.V. and maintained for an acclimatisation period of 1 week prior to the experiments, with 12 h light/dark cycles at a temperature of 25 ± 2°C and humidity of 55–80 %, with purified water and RodentChow food provided *ad libitum*. Animal care and maintenance were performed following the recommendations and guidelines described by the Internal Committee for the Care and Use of Laboratory Animals. The project was approved by the Bioethics Committee of the Escuela Nacional de Ciencias Biológicas of the Instituto Politécnico Nacional (ENCB-IPN), Registration Number ENCB/CEI/085/2023 Conbietica-09-CEI-002–20230327, and by the Research Ethics Committee of the Colegio de la Frontera Sur (Registration: CEI/2023/3798/05).

#### Model of hypertriglyceridaemia induced by Triton WR-1339 (tyloxapol)

The hypolipidaemic effect of AECS was evaluated as described by Salazar-Gomez *et al.* ^(21)^. Selected doses of AECS (150 and 600 mg/kg) were based on those of previous studies^(22)^, in which bioactive compounds (BC) -rich extracts were used, and a significant hypolipidaemic effect was demonstrated. This ensured that the doses were within a safety margin and were well below the reported acute lethality threshold (> 2000 mg/kg) for AECS, as established in previous research^(9)^. Animals were randomly assigned to four experimental groups (*n* 6), as follows: group I (negative control), group II (positive control, tyloxapol), group III (tyloxapol + AECS, 150 mg/kg) and group IV (tyloxapol + AECS, 600 mg/kg).

On day 0, groups III and IV were administered AECS intragastrically, while groups I and II received only the vehicle (Tween 80:water; 1:9, 10 ml/kg). Twenty-four hours after the intragastric administration, groups II, III and IV were given a dose of 400 mg/kg tyloxapol (Sigma-Aldrich CAT no. T8761) resuspended in isotonic sterile saline solution intraperitoneally, while group I (negative control) was given isotonic sterile saline solution at a dose of 5 ml/kg. At 24 and 48 h after administering tyloxapol, groups II, III and IV were again administered intragastrically with the extracts.

#### Collection and treatment of blood and tissue samples

Two hours after the final administration of the extracts, blood was collected by puncture of the retro-orbital sinus. These samples were centrifuged at 13 000 rpm for 15 min at 20°C, and the supernatant was recovered and stored in 1 ml Eppendorf microtubes at −70°C until analysis. The animals were euthanized by cervical dislocation and necropsied, and their livers were extracted. For OS analysis, the livers were cut into 0·5 g pieces with a scalpel and homogenised with 2 ml of cold saline phosphate buffer (pH 7·2). One part of the homogenate was separated for the determination of hepatic OS, and the other part was centrifuged at 13 000 rpm for 15 min at 4°C. Supernatants were separated from the precipitates and stored in 2 ml Eppendorf microtubes at −70°C until analysis^(23)^.

#### Evaluation of hepatic oxidative stress and serum hypertriglyceridaemia

The concentration of malondialdehyde (MDA) was measured as an indicator of the degree of lipoperoxidation using the thiobarbituric acid reactive substances technique. The total content of oxidised proteins was determined through the interaction of carbonylated proteins with 2,4-dinitrophenylhydrazine. Both of these measurements were performed in liver homogenates with no centrifugation, following the procedure described by Gutiérrez-Rebolledo *et al.* ^(23)^. The activity of antioxidant enzymes such as SOD (RANSOD CAT no. SD125) and GPx (RANSEL CAT no. RS504) was determined in the supernatants using RANDOX brand kits. Finally, the blood serum TAG concentration was determined using the procedure described in the commercial TRIS kit (CAT no. TR1697) (RANDOX Laboratories Ltd). All measurements were performed using UV-visible spectrophotometry on a Shimadzu UV-1700 Double Beam Scanning UV-Vis Spectrophotometer.

#### Proposal of a possible hypolipidaemic effect through in silico analysis

To evaluate hypolipidaemic activity by molecular docking, the twenty-five compounds identified in the Caco seed extract by HPLC-QTOF-MS analysis were selected. The chemical structures of these compounds were obtained from the PubChem platform (https://pubchem.ncbi.nlm.nih.gov/), while their molecular geometry was optimised using OpenBabel (MMFF94 with 20 000 steps). Furthermore, the Protein Data Bank (PDB) crystal structures of the following proteins were used as receptors: 3-hydroxy-3-methylglutaryl coenzyme A reductase (HMG-CoA) reductase (1HW8), PPAR*α* (3FEI), PPAR*γ* (2FVJ), PPAR*δ* (3PEQ) and lipoprotein lipase (LPL) (6OB0). The missing fragments of the proteins were reconstructed using SwissModel. The structures were prepared using the Dock Prep module of UCSF Chimera 1·14^(24)^, removing water molecules, lateral chains and ligands, adding hydrogens and assigning partial charges. The active site of each protein was defined based on the position of the co-crystallised ligand or controls: mevastatin for HMG-CoA reductase, (2S)-3-(4-{[2-(4-chlorophenyl)-1,3-thiazol-4-yl]methoxy}-2-methylphenyl)-2 ethoxypropanoic acid for PPAR*α*, isoquinolone for PPAR*γ*, phenoxyacetic acid for PPAR*δ* and, finally, 7-(3-cyano-4-hydroxyphenyl)-N-[2-(morpholin-4-yl) ethyl]dibenzo[b,f]oxepine-10-carboxamide for LPL. A grid box of sufficient size to accommodate each of the twenty-five ligands identified by UPLC-MS was applied to all structures. The grid box size and coordinates for each receptor were as follows: 1HW8, 40·72 × 42·52 × 45·22 Å (16·39, –19·37, 12·11); 3FEI, 37·30 × 31·72 × 37·48 Å (3·65, –0·48, 31·19); 2FVJ, 37·11 × 38·42 × 37. 25 Å (13·88, 64·48, 13·16); 3PEQ, 36·77 × 33·76 × 36·61 Å (3·39, 1·88, –11·18); and 6OB0, 25 × 25 × 25 Å (64·80, 0·00, 35·09). The AutoDock Vina software was used to perform the molecular docking and identify optimal binding conformations. The completeness of each run was 32, while the rest of the molecular docking parameters were kept at the preset values. After molecular docking, the interactions between the ligands and their respective receptors were analysed using Discovery Studio software. The molecular docking protocol for each receptor was validated by re-docking the co-crystalised ligand with the ligand conformation extracted from the PDB crystal and the Root Mean Square Deviation (RMSD) calculated in PyMOL. The RMSD value must be within a reliable range of 2 Å.

### Statistical analysis

Statistical programme Sigma Plot version 12 was used to determine the significance level (*P* ≤ 0·05) of the results through one-way ANOVA, with a Student Newman Keuls *post hoc* test.

## Results

### Quantification of phytochemical compounds in Caco seed extracts

The concentrations of the studied phytochemical components in AECS are presented in Table 1. For saponins and phytates, the extract presented values of 4730 ± 190 µg DGE and 1·0 ± 0·05 µg PAE/g, respectively. On the other hand, TI activity was 14 ± 0·08 Trypsin Inhibitor Units (TIU).

**Table 1.** Bioactive compounds identified in the aqueous extract of Caco seed using HPLC-QTOF-MS Table 1 long description.

	Concentration	
	Mean	sd
Saponin (µg DGE/g sample)	4730	190
PC (µg PAE/g sample)	1·0	0·05
Trypsin inhibitors (TIU)	6·14	0·08

HPLC-QTOF-MS, HPLC-quadrupole time-of-flight-MS; DGE, diosgenin equivalent; PC, phytate concentration; PAE, phytate acid equivalent.

Data are shown as mean values (standard deviation (sd)).

### Metabolomic analysis by HPLC coupled to electrospray ionisation quadrupole time-of-flight MS

**Figure 1.**
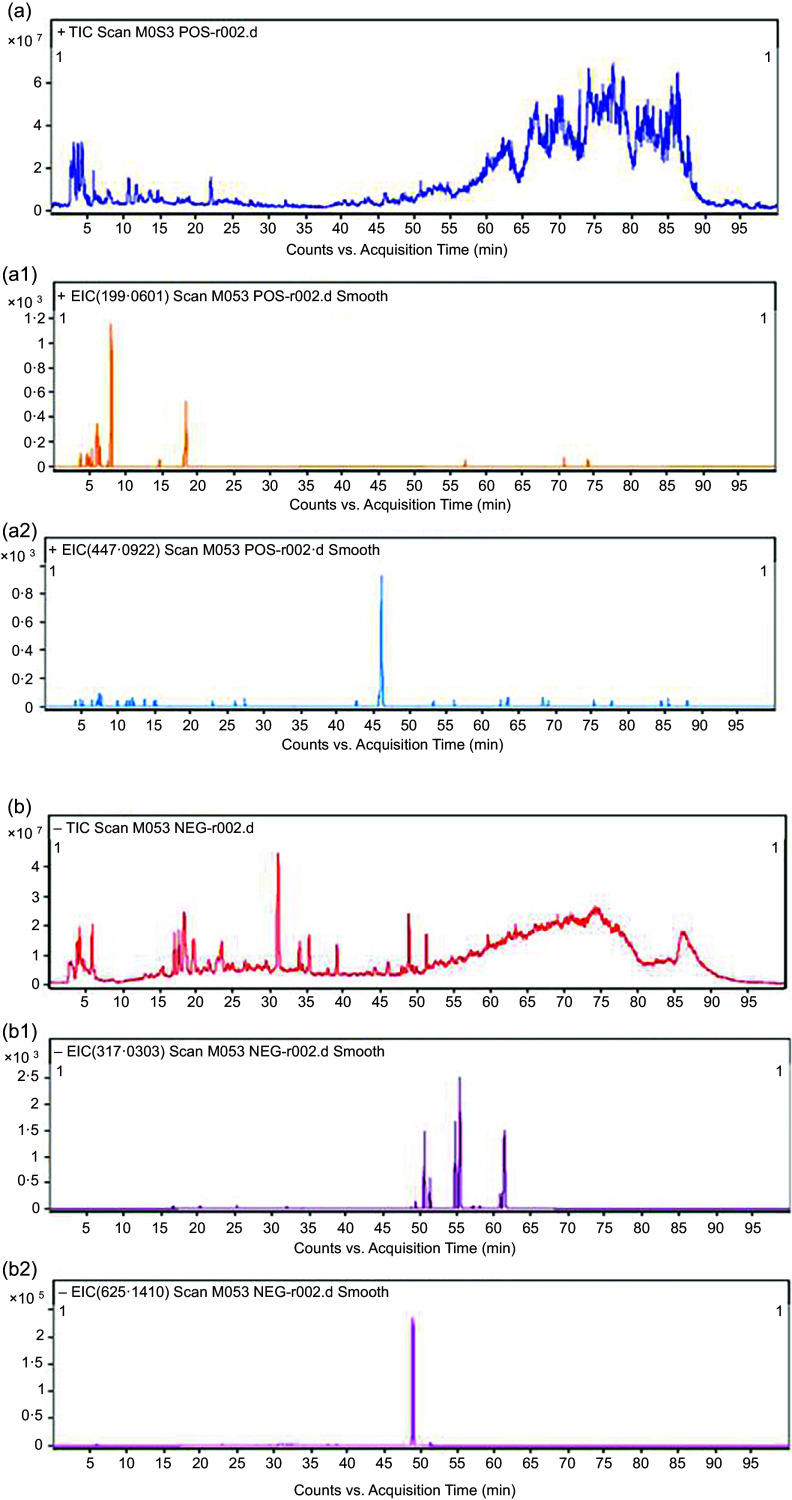
Chromatogram of phenolic compounds identified in the positive (a) and negative (b) modes from defatted *C. icaco* seed extract. 3,4-O-Dimethylgallic acid (a1), apigenin 7-O-glucuronide (a2), myricetin (b1) and myricetin 3-O-rutinoside (b2).

Table 2 shows the main BC in AECS as measured by LC-ESI-QTOF-MS/MS. Compounds were detected in a mass range (*m/z*) of 50–500 Da and with a mass error of < 2. Twenty-five formulas were identified in the extract. In positive mode, the following were found: C_9_H_10_O_15_, C_21_H_18_O_11_, C_15_H_10_O_7_ and C_15_H_14_O. In negative mode, the following were identified: C_33_H_40_O_20_, C_14_H_6_O_8_, C_7_H_6_O_3_, C_13_H1_6_O_8_, C_7_H_6_O_5_, C_13_H_17_O_10_, C_15_H_14_O_6_, C_9_H_8_O_3,_ C_15_H_18_O_8_, C_18_H_17_NO_5_, C_16_H_18_O_10_, C_7_H_6_O_4_, C_15_H_10_O_7_, C_27_H_30_O_17_, C_16_H_20_O_10_, C_21_H_20_O_13_, C_20_H_18_O_12_, C_21_H_18_O_14_, C_27_H_30_O_16_, C_21_H_20_O_12_ and C_15_H_10_O_8_. These formulas correspond to eleven phenolic acids and thirteen flavonoids. It can therefore be inferred that these are the main compounds present in AECS. These compounds were selected based on retention time (Tr), accuracy (expressed by the error) and peak signal intensity in both modes ([M – H]–/[M + H]+), measured in counts per second (cps). The Tr values of the compounds identified in AECS were between 7 and 55·5 min. Figure 1 shows a fragment of the chromatogram of the phenolic compounds identified in the positive (a) and negative (b) modes from AECS and features 3,4-O-dimethylgallic acid (a1), apigenin 7-O-glucuronide (a2), myricetin (b1) and myricetin 3-O-rutinoside (b2).

**Table 2.** Concentrations of the phytochemical compounds studied in the aqueous extract of Caco seed Table 2 long description.

Number	rT (min)	Theoretical *m/z*	Measured *m/z*	Error (amu)	Signal intensity (cps)	Formula	Compounds
	Positive mode						
1	8	199·1700	199·0601	–0·1099	1·1×10^3^	C_9_H_10_O_15_	3,4-O-dimethylgallic acid
2	22	307·2700	307·0813	–0·1887	6·0×10^4^	C_21_H_18_O_11_	(−)-Epigallocatechin
3	46	447·4000	447·0922	–0·3078	0·9×10^3^	C_15_H_10_O_7_	Apigenin 7-O-glucuronide
4	46·5	307·2600	303·0500	–4·2100	6·0×10^4^	C_15_H_14_O	5,6,7,3,4 Pentahydroxyisoflavone
	Negative mode						
5	4	755·6587	755·2040	–0·4547	3·5×10^3^	C_33_H_40_O_20_	Kaempferol 3-O-glucosylrhamnosyl-galactoside
6	7	301·1900	300·9990	–0·1910	3·8×10^2^	C_14_H_6_O_8_	Ellagic acid
7	18	137·1200	137·0244	–0·0956	1·0×10^5^	C_7_H_6_O_3_	p-hydroxybenzoic acid
8	18·8	299·2600	299·0772	–0·1828	6·5×10^4^	C_13_H1_6_O_8_	4-Hydroxybenzoic acid 4-O-glucoside
9	22·5	169·1200	169·0142	–0·1058	5·5×10^3^	C_7_H_6_O_5_	Gallic acid
10	22·5	331·2600	331·0670	–0·1930	1·3×10^4^	C_13_H_17_O_10_	Gallic acid 4-O-glucoside
11	29·5	289·2700	289·0717	–0·1983	1·52×10^3^	C_15_H_14_O_6_	Epicatechin
12	30	163·0473	163·0401	–0·0072	6·2×10^4^	C_9_H_8_O_3_	Coumaric acid
13	30	325·3000	325·0929	–0·2071	2·5×10^4^	C_15_H_18_O_8_	p-Coumaric acid 4-O-glucoside
14	30	326·3000	326·1034	–0·1966	2·4×10^3^	C_18_H_17_NO_5_	p-Coumaroyl tyrosine
15	30	369·3100	369·0827	–0·2273	4·3×10^2^	C_16_H_18_O_10_	Ferulic acid 4-O-glucuronide
16	41	153·1200	153·0193	–0·1007	6·0×10^3^	C_7_H_6_O_4_	Protocatechuic acid
17	44	301·2300	301·0354	–0·1946	6·3×10^3^	C_15_H_10_O_7_	Quercetin
18	48	625·5000	625·1410	–0·3590	2·5×10^5^	C_27_H_30_O_17_	Myricetin 3-O-rutinoside
19	49	371·3200	371·0983	–0·2217	0·9×10^5^	C_16_H_20_O_10_	Dihydroferulic acid 4-O-glucuronide
20	49	479·4000	479·0831	–0·3169	4·7×10^4^	C_21_H_20_O_13_	Myricetin 3-O-glucoside
21	50	449·3000	449·0725	–0·2275	4·1×10^3^	C_20_H_18_O_12_	Myricetin 3-O-arabinoside
22	51	491·0624	493·0624	2·0000	2·55×10^4^	C_21_H_18_O_14_	Myricetin 3-O-glucoronide
23	51	609·5000	609·1461	–0·3539	4·0×10^4^	C_27_H_30_O_16_	Kaempferol 3,7-O-diglucoside
24	52	463·4000	463·0882	–0·3118	1·25×10^5^	C_21_H_20_O_12_	Myricetin 3-O-rhamnoside
25	55·5	317·2300	317·0303	–0·1997	2·5×10^3^	C_15_H_10_O_8_	Myricetin

Note: Error (amu) was calculated as an absolute value.

### Serum lipid profile and hepatic oxidative stress

Serum TAG levels in male CD1 mice administered with tyloxapol (group II) increased up to 4·34 mmol/dl (*P* < 0·05) relative to those in group I (negative control), which had values of 1·27 ± 0·04 mmol/dl. This represented a more than threefold increase in content. On the other hand, after the period of administration of the extracts, decreases of blood TAG of 3 and 64 % were observed in groups III and IV, respectively, compared with the damage control group with no treatment (4·34 ± 0·40 mmol/l), which was almost at the same level as those of the healthy animals or negative control group (Table 3).

**Table 3.** Effect of aqueous extract of Caco seeds on serum TAG levels and biomarkers of hepatic oxidative stress in male CD1 mice with hypertriglyceridaemia Table 3 long description.

	TAG (mmol/dl serum)		LPO (mmol MDA/g tissue)		POx (mmol CO●/g tissue)		SOD (IU/g tissue)		GPx (IU/g tissue)	
Groups	Mean	se	Mean	se	Mean	se	Mean	se	Mean	se
I	1·27	0·04	39·52	2·6	253·12	15·9	688	53·9	263·05	45·9
II	4·34	0·40^a^	45·35	1·1^a^	336·55	8·4^a^	986·72	42·3^a^	532·30	68·1^a^
III (150)	4·21	0·57^a^	42·55	2·5^a^	316·55	18·5^a^	701·63	76·5^b^	324·24	38·2^b^
IV (600)	1·56	0·18^a,b,c^	29·78	2·0^b,c^	306·08	19·3^a,b^	645·48	55·7^b^	686·15	87·2^a,c^

LPO, lipoperoxidation; MDA, malondialdehyde; POx, oxidised protein; CO, carbonyl group; SOD, superoxide dismutase; GPx, glutathione peroxidase.

Data are presented as mean values (standard error of the mean (se)). Treatments were administered intragastrically every day for 3 d (groups II, III and IV). On day 1, 1 h after the intragastric treatment, the mice received a dose of tyloxapol (400 mg/kg resuspended in isotonic sterile saline solution) intraperitoneally. Statistical analysis: one-way ANOVA and Student–Newman–Keuls *post hoc* test (*P* ≤ 0·05); a *v*. I; b *v*. II; c *v*. III; (*n* 6).

A significant decrease in lipoperoxidation and oxidised proteins was observed following treatment with the extract at different doses. Group IV presented a lower degree of lipoperoxidation in the liver tissue (29·8 ± 2·0 mmol of MDA/g tissue) compared with group III, which presented a content of 42·55 ± 2·5 mmol of MDA/g tissue, corresponding to an inhibition of lipid oxidation of 6·2 %. This value was statistically equal to that of the positive control group (45·4 ± 1·1 mmol of MDA/g tissue) (Table 3). In the case of oxidised proteins, groups III and IV presented 316·55 ± 18·5 and 306·08 ± 19·3 mmol of carbonyl groups (CO)/g tissue, respectively, corresponding to an inhibition of protein oxidation of 10 and 9 %, respectively. These values were statistically equal to those of the positive control group (336·5 ± 8·4 mmol of CO/g tissue) (Table 3).

The other biomarkers that were evaluated to establish the *in vivo* antioxidant activity for both extracts at different doses were the antioxidant enzymes SOD and GPx. The SOD enzyme increased its antioxidant activity by 29 % (701·63 ± 76·5 IU/g tissue) and 35 % (645·48 ± 55·7 IU/g tissue) in groups III and IV, respectively, being statistically equal to the negative group (688 ± 53·9 IU/g tissue). The highest antioxidant activity of GPx was presented in group IV, with 47·2 % compared with that of the positive control group (532·3 ± 68·1 IU/g tissue) (Table 3). Administration of both extracts caused a reversal of these values, which then approached the levels observed in the negative control group (group I).

### Proposal of a possible mechanism of hypolipidaemic action

To determine the possible mechanism of action of AECS, molecular docking analysis was performed with the twenty-five identified compounds against five targets of interest related to lipid metabolism (HMG-CoA reductase, PPAR*α*, PPAR*γ*, PPAR*δ* and LPL). The software AutoDock Vina was used to determine the energy of affinity between the ligands and the proteins in question (Table 4).

**Table 4.** Binding affinities between the aqueous extract of Caco seed compounds and proteins related to lipid metabolism Table 4 long description.

Compound	HMG-CoA reductase	PPAR*α*	PPAR*γ*	PPAR*δ*	LPL
3,4-O-Dimethyl gallic acid	–5·9	–6·3	–6·2	–6·1	–5·7
4-Hydroxybenzoic acid 4-O-glucoside	–7·4	–7·7	–7·3	–8·0	–6·3
5,6,7,3,4-Pentahydroxy isoflavone	–8·2	–8·5	–8·1	–8·6	–7·8
Apigenin-7-O-glucuronide	–9·3	–9·5	–9·2	–10·0	–8·4
Dihydroferulic acid 4-O-glucuronide	–7·6	–8·0	–7·8	–8·4	–6·7
Ellagic acid	–9·2	–7·8	–8·9	–8·3	–7·7
Epicatechin	–8·5	–7·8	–7·9	–8·5	–7·8
(−)-Epigallocatechin	–8·3	–7·5	–7·6	–8·4	–7·2
Ferulic acid 4-O-glucuronide	–7·9	–8·4	–7·7	–8·3	–7·0
Gallic acid	–6·4	–5·9	–5·9	–5·8	–6·0
Gallic acid 4-O-glucoside	–7·5	–7·8	–7·1	–7·9	–6·2
Kaempferol 3-O-glucosylrhamnosyl galactoside 1	–9·0	–6·2	–6·4	–7·8	–7·9
Kaempferol-3,7-O-diglucoside 1	–8·3	–8·3	–8·4	–9·1	–8·0
Myricetin	–8·7	–7·9	–8·2	–8·1	–7·7
Myricetin 3-O-arabinoside	–7·4	–7·8	–7·6	–8·3	–6·9
Myricetin 3-O-glucoside	–8·2	–8·0	–7·8	–8·3	–7·1
Myricetin 3-O-glucuronide	–8·5	–8·4	–8·0	–8·5	–7·2
Myricetin 3-O-rhamnoside	–8·4	–6·8	–7·6	–8·1	–8·1
Myricetin 3-O-rutinoside	–9·0	–9·2	–6·9	–10·2	–7·6
N-p-Coumaroyltyrosine	–8·0	–8·1	–8·4	–8·6	–7·7
P-Coumaric acid	–6·5	–6·6	–6·5	–6·0	–6·4
P-Coumaric acid4-O-glucoside	–7·4	–7·6	–7·5	–8·0	–6·9
P-hydroxybenzoic acid	–5·9	–5·9	–5·8	–5·4	–5·6
Protocatechuic acid	–6·4	–6·2	–6·0	–5·8	–6·0
Quercetin	–8·7	–7·7	–8·4	–8·5	–7·6
Control^*^	–7·5	–7·0	–9·4	–9·7	–10·8

LPL, lipoprotein lipase.

* Mevastatin for HMG-CoA reductase, (2S)-3-(4-{[4-{[2-(4-chlorophenyl)-1,3-thiazol-4-yl]methoxy}-2-methylphenyl)-2-ethoxypropanoic acid for PPAR*α*, isoquinolone for PPAR*γ*, phenoxyacetic acid for PPAR*δ* and 7-(3-cyano-4-hydroxyphenyl)-N-[2-(morpholin-4-yl) ethyl]dibenzo [b,f] oxepine-10-carboxamide for LPL.

Molecular docking results suggest that AECS compounds could bind to enzymes responsible for lipid metabolism and thus favour the observed reduction of their concentrations *in vivo* (Table 3). The compounds with the greatest affinities (lower than −7·6 kcal/mol) for the five targets evaluated were 5,6,7,3,4-pentahydroxy isoflavone, apigenin-7-O-glucuronide, ellagic acid, epicatechin, kaempferol-3,7-O-diglucoside 1, myricetin, myricetin 3-O-glucoside, myricetin 3-O-glucuronide, myricetin 3-O-rutinoside, N-p-coumaroyltyrosine and quercetin.

**Figure 2.**
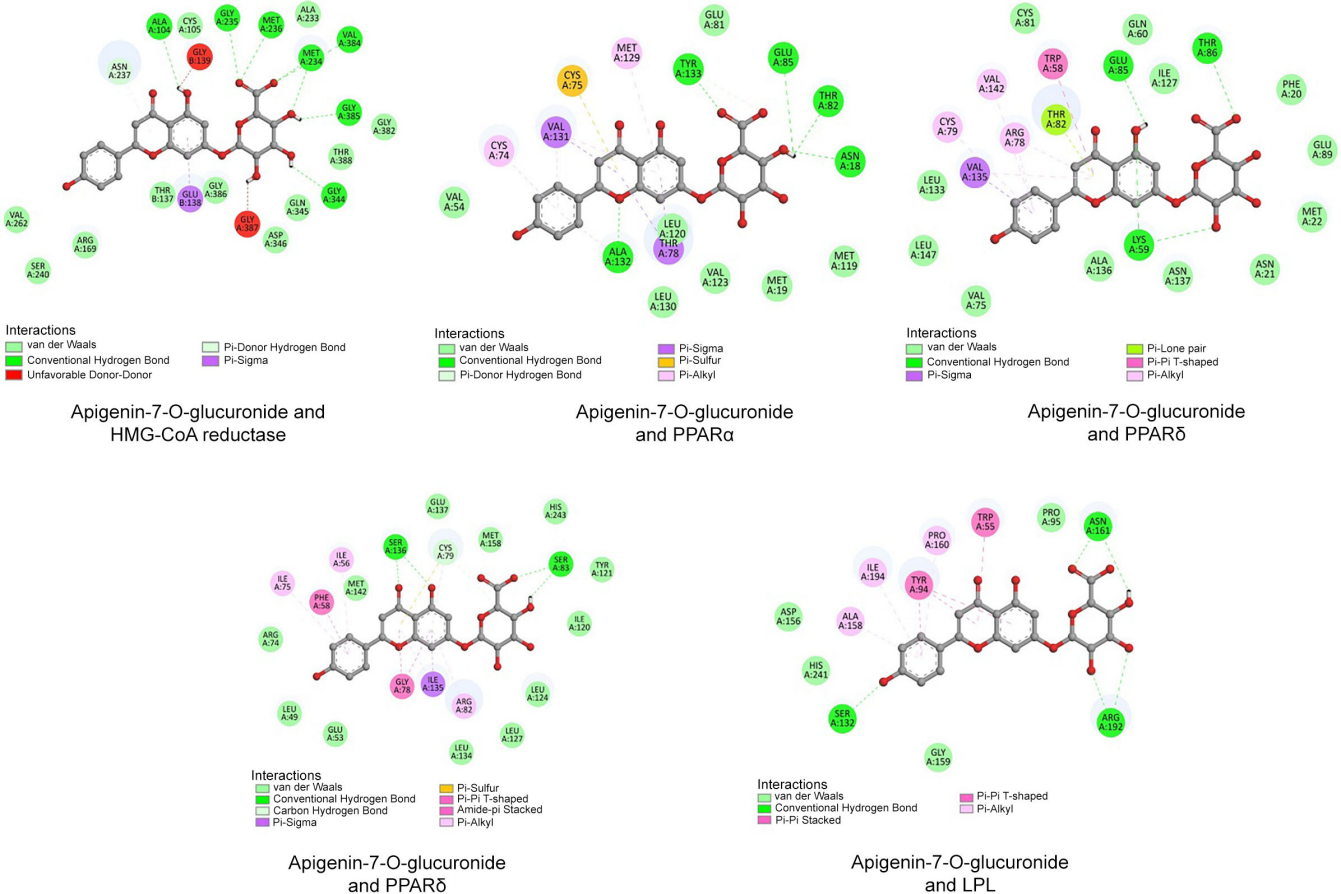
Interactions of the compound apigenin-7-O-glucuronide with five target molecules related to lipid metabolism (HMG-CoA reductase, PPAR*α*, PPAR*γ*, PPAR*δ* and lipoprotein lipase (LPL)).

Likewise, the apigenin-7-O-glucuronide ligand presented the most favourable results, with binding energy values below −8·4 kcal/mol, while myricetin 3-O-rutinoside also exhibited favourable energies, but only with the targets HMG-CoA reductase, PPAR*α* and PPAR*δ* (Figure 2).

Table 5 shows the bioactive compounds (BC) with the best binding affinities and the amino acids to which they bind. To a large extent, AECS compounds bind to the same residues of the target proteins as the compounds that were found in the PDB archive crystal, with the most prominent being apigenin-7-O-glucuronide.

**Table 5.** Bioactive compounds in aqueous extract of Caco seeds with the greatest binding energies to their receptors and the amino acid residues with which they interact Table 5 long description.

Compound/1HW8	Energy	Residues
Apigenin 7-O-glucuronide	–9·3	Ala104, Cys105, Thr137, Glu138, Gly139, Ala233, Met234, Gly235, Met236, Asn237, Gly344, Val 384, Gly385, Gly386, Gly387
Myricetin	–8·7	Glu138, Gly139, Cys140, Arg169, Met236, Ser263, Asn265, Lys271, Lys314, Ala330, Leu432
Myricetin 3-O-arabinoside	–7·4	Glu138, Gly139, Cys140, Arg169, Ser263, Lys271, Lys314, Ala330, Leu432
Myricetin 3-O-glucoside	–8·2	Ala104, Cys105, Glu138, Ala233, Met234, Gly235, Met236, Asn237, Gly385, Gly386
Myricetin 3-O-glucuronide	–8·5	Ala104, Cys105, Glu138, Gly139, Arg169, Met234, Gly235, Met236, Asn237, Val384, Gly385, Gly386, Gly387, Asn389
Myricetin 3-O-rhamnoside	–8·4	Glu138, Gly139, Cys140, Arg169, Glu244, Val262, Ser263, Asn265, Lys271, Lys314, Ala330, Asn334
Myricetin 3-O-rutinoside	–9·0	Ala104, Cys105, Glu138, Gly139, Ala233, Met234, Gly235, Met236, Met238, Val384, Gly385, Gly386
Crystalised ligand (control)	–7·5	Glu138, Cys140, Leu141, Arg169, Met236, Val262, Ser263, Asp269, Lys270, Lys271, Lys314, His331, Asn334, Leu432, Leu436
Compound/PPAR*α*		
Apigenin 7-O-glucuronide	–9·5	Asn18, Met19, Val54, Cys74, Cys75, Thr78, Thr82, Glu85, Leu120, Met129, Val131, Ala132, Tyr133
Myricetin	–7·9	Asn18, Met19, Cys75, Thr78, Glu85, Met119, Leu120, Val123, Val131, Ala132, Tyr133
Myricetin 3-O-arabinoside	–7·8	Ala49, Val54, Cys74, Cys75, Thr78, Met129, Val131, Ala132, Tyr133, Ile138, Leu143, Met154
Myricetin 3-O-glucoside	–8·0	Ala49, Val54, Cys74, Cys75, Cys77, Thr78, Val131, Ala132, Tyr133
Myricetin 3-O-glucuronide	–8·4	Ala49, Leu53, Val54, Cys74, Cys75, Cys77, Thr78, Met129, Val131, Ala132, Tyr133
Myricetin 3-O-rhamnoside	–6·8	Ala240, Gln241, Gln244, Tyr263, Arg264, Asp265, Met266, Tyr267
Myricetin 3-O-rutinoside	–9·2	Asn18, Met19, Ile40, Ala49, Val54, Cys74, Cys75, Cys77, Thr78, Thr82, Val131, Ala132, Tyr133, Ile138
Crystalised ligand (control)	–7·0	Phe72, Cys74, Cys75, Thr78, Ser79, Tyr113, Leu120, Met129, Val131, Ile153, Met154, His 239, Tyr263
Compound/PPAR*γ*		
Apigenin 7-O-glucuronide	–9·2	Ile56, Phe58, Ile75, Gly78, Cys79, Arg82, Ser83, Leu124, Ile135, Ser136, Met158
Myricetin	–8·2	Lys57, Gly78, Cys79, Phe81, Arg82, Glu85, Ile135, Ser136, Glu137
Myricetin 3-O-arabinoside	–7·6	Leu247, Ile250, Lys251, Met257, Ser258, Leu259, Gln264, Lys268, Tyr267
Myricetin 3-O-glucoside	–7·8	Ile250, Lys251, Met257, Ser258, Leu259, Gln264, Lys268
Myricetin 3-O-glucuronide	–8·0	Leu247, Ile250, Lys251, Ser258, Gln264, Lys268
Myricetin 3-O-rhamnoside	–7·6	Leu247, Ile250, Lys251, Ser258, Leu259, Gln264, Tyr267
Myricetin 3-O-rutinoside	–6·9	Val244, Leu247, Gln248, Lys251, Leu259, Gln264, Tyr267, Lys268
Crystalised ligand (control)	–9·4	Ile75, Phe76, Cys79, Ser83, Phe154, Phe157, Met158, His243, Tyr267
Compound/PPAR*δ*		
Apigenin 7-O-glucuronide	–10·0	Met22, Trp58, Lys59, Val75, Arg78, Cys79, Thr82, Glu85, Thr86, Ile127, Val135, Ala136, Asn137, Val142
Myricetin	–8·1	Met22, Trp58, Lys59, Thr82, Glu85, Thr86, Ile127, Asn137
Myricetin 3-O-arabinoside	–8·3	Met22, Thr82, Thr86, Glu89, Met123, Leu124, Ile127
Myricetin 3-O-glucoside	–8·3	Met22, Lys59, Cys79, Thr82, Thr86, Glu89, Met123, Leu124, Ile127, Ala136, Asn137
Myricetin 3-O-glucuronide	–8·5	Met22, Lys59, Thr82, Glu85, Thr86, Glu89, Met123, Leu124, Ile127, Asn137
Myricetin 3-O-rhamnoside	–8·1	Met22, Cys79, Thr86, Glu89, Thr82, Ile120, Met123, Leu124, Ile127, Ala136, Asn137
Myricetin 3-O-rutinoside	–10·2	Met22, Lys59, Arg78, Cys79, Thr82, Thr86, Glu89, Ile120, Leu124, Ile127, Leu133, Val135, Ala136, Asn137, Val142
Crystalised ligand (control)	–9·7	Met22, Trp58, Lys59, Arg78, Cys79, Thr82, Glu85, Thr86, Met123, Ser126, Ile127, Leu134, Val135, Asn137, Val142
Compound/lipoprotein lipase		
Apigenin-7-O-glucuronide	–8·4	Trp55, Tyr94, Pro95, Ser132, Ala158, Pro160, Asn161, Arg192
Kaempferol 3-O-glucosylrhamnosyl galactoside 1	–7·9	Trp55, Tyr94, Pro95, Ser132, Asn161, Arg192, Ile194, Gln235
Kaempferol-3,7-O-diglucoside 1	–8·0	Trp55, Tyr94, Gln235, Ala158, Gly159, Pro160, Asn161, Arg192, Ile194
Myricetin 3-O-rhamnoside	–8·1	Trp55, Tyr94, Pro160, Ile194, Gln235, Leu236, His241
Crystalised ligand (control)	–10·8	Trp55, His93, Tyr94, Pro95, Ala158, Pro160, Ile194, Lys238, His241

## Discussion

Plant-based foods contain significant concentrations of secondary metabolites, also known as BC. Although these are not considered nutrients, their presence helps strengthen the body’s defense system, acting mainly as antioxidants^(12)^. Moderate consumption of these metabolites is recommended since excess amounts could be harmful to the body, acting as antinutrients^(25)^. The BC in plants that have been the subject of most study are the alkaloids, flavonoids, tannins and phenolic compounds. However, there are other BC of biological importance, such as cardiac glycosides, haemagglutinin, oxalates, phytates and saponins, among others^(26)^. Despite being considered antinutrients, these metabolites play a regulatory role in organisms through various biological activities depending on their concentration, for example, their saponin content, which was higher for AECS (4730 ± 190 μg DGE/g sample) than that reported by Salawu *et al.* ^(27)^ for the tropical almond (*Terminalia catappa*) and sweet almond (*Prunus amygdalus*), in which saponin concentrations of 0·086 and 0·0158 μg/g were reported, respectively. Although these results do not pertain to the seeds of closely related species, they are relevant because they are used for human consumption, are botanically close (hard woody pericarp) and have a rich fat content^(28)^. Although the concentration of saponins was higher, it has been reported that aqueous extracts of Caco seeds are potentially safe, with a median lethal dose > 2 g/kg by the intragastric route reported in an acute toxicity study in male CD1 mice^(9)^.

Another crucial antinutrient that requires evaluation is phytic acid, which forms during seed maturation and represents 60–90 % of its total phosphate. Thus, its quantification furthers our understanding of seed quality and viability. In this context, the phytate concentration in AECS was 1·0 ± 0·05 μg PAE/g sample, which represents 0·0001 %. Phytate concentration values have been reported in oilseeds such as sunflower, sesame and soybean (1·0–5·4 % dry weight) and nuts such as hazelnuts, walnuts and almonds (0·1 and 9·4 % dry weight)^(29)^. These reported values are higher than those found in this study. Although it has been reported that the presence of this compound decreases the bioavailability of minerals (Ca^2+^, Zn^2+^ and Fe^2+^), as well as the solubility, functionality and digestibility of proteins and carbohydrates, health benefits have been reported as a result of the chelation of toxic metals such as cadmium (Cd) and palladium (Pd)^(30)^. Several factors influence the dietary intake of phytic acid in humans, which can be 1000–2000 mg/kg of body weight (a value higher than that found in this seed), with no adverse health effects. This intake can vary depending on the amount of plant-based foods consumed, food processing, location (countries, rural and urban areas), sex and age^(31)^.

Finally, as part of nutritional quality evaluation, the activity of TI, an enzyme belonging to proteases important to the nutrition of many animals, including humans, was measured. When inhibited by chemicals present in plant foods (legumes, cereals and oilseeds), its proteolytic activity decreases in the gastrointestinal tract, impeding the digestion of proteins and absorption of minerals, particularly Ca, Zn and Fe^(30,32)^. The amount of chemical substances present in food can be measured indirectly by the inhibition of trypsin activity and is expressed as TIU per gram of sample, which for AECS was 6·14 ± 0·08, which is lower than that reported for *Jatropha curcas* seeds (8347 TIU/g sample)^(19)^. These values are important and demonstrate the viability of the seeds for consumption since the presence of high concentrations of phytates and TI can compromise nutrient and micronutrient absorption.

Beyond their quantification as antinutritional factors, it is essential to analyse the contribution of these BC to the pharmacological action mechanism of AECS. In this context, the antioxidant potential of AECS is not only a function of its phenolic compounds^(9)^ but also of its interaction with other BC. The high concentration of saponins (4730 ± 190 μg DGE/g) plays an essential regulatory role by mitigating OS and increasing endogenous antioxidants (SOD and GPx), which reinforces antioxidant endogenous defense^(33,34)^. In contrast, although phytates (1·0 ± 0·05 µg PAE/g) and TI (6·14 ± 0·08 TIU/g) are present at low levels, their quantification is key to confirming the safety of AECS. The phytates likely provide a secondary antioxidant benefit through the chelation of transition metals, thereby inhibiting the production of reactive oxygen species^(35)^. Overall, the balance between the direct antioxidant potential of polyphenols and the strengthening of endogenous defences provided by saponins, together with the safety of the total antinutrient content of AECS, explains its robust efficacy and therapeutic effect.

Although the biological activity of AECS can be partially explained by the quantification of known BC (saponins, phytic acid and TI), this quantitative approach does not encompass all the phytochemicals present in the extract. Therefore, based on preliminary results that reported significance in Total phenolic compounds (TPC)^(9)^, structural identification of these other BC responsible for the observed biological activity was conducted with an untargeted metabolomic profile using HPLC-QTOF-MS. This analysis allows for the efficient separation of CB, providing exact mass data and fragmentation patterns, which are essential for the identification of a wide range of BC, including those present in trace concentrations that could contribute to the observed biological efficacy^(36)^.

Among the phenolic acids, hydroxybenzoic (3,4-O-dimethylgalic acid, ellagic acid, *p*-hydroxybenzoic acid, 4-hydroxybenzoic acid 4-O-glucoside, gallic acid, gallic acid 4-O-glucoside, coumaric acid, p-coumaric acid 4-O-glucoside and protocatechuic acid) and hydroxycinnamic (p-coumaroyl tyrosine, ferulic acid 4-O-glucuronide and dihydroferulic acid 4-O-glucuronide) acids were identified. The compound 3,4-*O*-dimethylgalic acid (compound 1) was detected in positive mode in AECS. This compound is a major derivative of gallic acid and has been reported in hydroalcoholic extracts of date palm (*Phoenix dactylifera* L.) seeds^(37)^. Compound 6 has been identified in negative mode in hydroalcoholic extracts obtained from *C. icaco* pulp and peel^(38)^, while compounds 22 and 24 have been found in aqueous extracts of *C. icaco* leaves obtained by infusion^(39)^.

Another family of phenolic compounds present in AECS was that of flavonoids, including epigallocatechin, apigenin 7-O-glucuronide, 5,6,7,3,4 pentahydroxyisoflavone, kaempferol 3-O-glucosylramnosyl-galactoside, epicatechin, quercetin, myricetin 3-O-ruthinoside, myricetin 3-O-glucoside, myricetin 3-O-arabinoside, myricetin 3-O-glucuronide, kaempferol 3,7-O-diglucoside, myricetin 3-O-rhamnosyl-galactoside and myricetin. Bastos Silva *et al.* ^(40)^ reported that Caco leaf extract produced an increase in peak area at Tr of 15·59 and 23·14 min, confirming the presence of flavonoids such as rutin and myricetin. In another study by Farid *et al.* ^(41)^, the presence of 7-O-glucuronide of apigenin was identified in aqueous extracts of *Anabasis aretioides* with a Tr (40 min) lower than that obtained in this study (46 min). These results demonstrate that some polyphenols identified in this study are present within the Chrysobalanaceae family^(11,14–16,38–40,42)^, are a main source of antioxidants and have a beneficial effect on lipid metabolism^(43,44)^.

The HPLC-QTOF-MS analysis identified a broad spectrum of chemical biomarkers in AECS, with a predominance of phenolic acids (gallic, ellagic and *p*-coumaric, among others) and flavonoids (quercetin, epicatechin, myricetin, apigenin and their derivatives)^(9)^. Given the well-documented biological activities of these polyphenols, their potential molecular mechanisms of action were postulated and evaluated. *In silico* approaches were used to predict pharmacokinetic and pharmacodynamic properties and to explore ligand–target interactions that could explain the *in vivo* effects of the extract^(45)^.

The tyloxapol model reproduces a pathophysiological state characterised by impaired lipid clearance due to LPL inhibition and consequent hepatic lipid overproduction (lipogenesis), a combination that underlies dyslipidaemia and non-alcoholic fatty liver disease^(4–7)^. Tyloxapol induces acute hyperlipidaemia primarily through LPL inhibition^(21,46–49)^, making this model appropriate for evaluating the AECS capacity to reverse enzymatic inhibition and restore lipid homeostasis. In this model, AECS at 600 mg/kg produced a significant reduction in plasma TAG (1·56 mmol/l), whereas 150 mg/kg was ineffective. This outcome parallels previous reports linking high tannin and flavonoid content in aqueous plant (*Ocimum basilum*) extracts with TAG reductions in rodents at 500 mg/kg^(48)^. This result may reflect the antioxidant, anti-inflammatory and hypolipidaemic capacity of the BC present in the extract^(50)^.

The dose-dependent *in vivo* hypolipidaemic effect observed with AECS is compatible with the predicted high affinity of glycosylated flavonoids for HMG-CoA reductase and PPAR^(51–54)^, which would be expected to reduce hepatic lipogenesis and enhance fatty acid catabolism.

Molecular docking predicted differential binding profiles for flavonoids and phenolic acids against pharmacologically relevant targets for lipid homeostasis. Glycosylated flavonoids, such as apigenin-7-O-glucuronide, showed higher predicted binding affinities for the active sites of HMG-CoA reductase and peroxisome proliferator-activated receptors (PPAR*α*, PPAR*δ*, PPAR*γ*)^(51–54)^, as well as for LPL. These results are consistent with reports that glycosylation (glucose, galactose, arabinose, rhamnose) and positional changes of the hydroxyl groups modulate flavonoid bioactivity and pharmacological properties^(50,54)^.

In contrast, several phenolic acids (*p*-hydroxybenzoic acid, 3,4*O*-dimethyl gallic acid, protocatechuic acid) exhibited low predicted affinity for the LPL active site, suggesting that these compounds are unlikely to act as potent competitive inhibitors of LPL^(55)^. Low active-site affinity does not exclude biological activity, but it may indicate allosteric modulation or indirect effects such as transcriptional induction of LPL expression^(56)^. Therefore, the modulatory role of these phenolic acids in LPL catalytic activity requires validation *in vitro*.

AECS enhances the hyperlipidaemic effect through the stimulation of LPL and/or fatty acid beta-oxidation, likely due to their BC. Arce-Ortiz *et al.* ^(9)^ reported the content of total phenols, flavonoids and condensed tannins in AECS, with *in vitro* antioxidant potential and anti-inflammatory effects in *in vivo* models. These compounds could be responsible for the reduction of TAG and are associated with a significant decrease in atherosclerosis, thereby reducing the risk of heart attacks, strokes and death^(50)^.

On the other hand, an experimental hypertriglyceridaemia assessment of hepatic OS biomarkers confirmed a marked redox imbalance in hyperlipidaemic animals: MDA and increased protein carbonyls in the hepatic tissue of tyloxapol-induced and un-treated mice, while activities of antioxidant enzymes (SOD, GPx) increased, likely reflecting compensatory responses to elevated reactive oxygen species and reactive nitrogen species, as occurs in the human pathophysiology of these dyslipidaemias and CVD^(57,58)^.

AECS administration (150 and 600 mg/kg) attenuated lipid and protein oxidative damage, significantly lowering MDA and carbonyl levels; SOD activity returned to levels similar to those of vehicle-treated controls, and GPx activity normalised only at 600 mg/kg. The *in vivo* antioxidant protection aligns with the polyphenolic profile of the extract: quercetin, apigenin, myricetin and their derivatives act as potent radical scavengers (azino-bis(3-ethylbenzothiazoline-6-sulfonic acid) (ABTS), 2,2-diphenyl-1-picrylhydrazyl (DPPH)) and metal-reducing agents (Ferric Reducing Antioxidant Power (FRAP)), validating *in vitro* antioxidant assays^(9)^. At the molecular level, the molecular docking results support direct interactions of these flavonoids with PPAR and HMG-CoA reductase, suggesting a possible mechanistic link between antioxidant capacity and modulation of lipid metabolism^(51–54)^.

Complementary BC present at low concentrations, such as phytates and saponins, likely contribute to the overall effect without exceeding safety margins^(31)^. Phytate chelation of transition metals can reduce metal-catalysed radical formation, offering a secondary antioxidant benefit, while saponins have been reported to modulate lipid metabolism and to enhance endogenous antioxidant defences (increased hepatic SOD and GPx) and to attenuate proinflammatory mediators (IL 6, TNF-*α*)^(30,31)^. *In vivo* evidence that phytic acid supplementation reduces serum TAG and raises HDL-cholesterol supports a contributory role for phytates in lipid modulation^(32,35)^.

Enzymatic and antioxidant effects observed *in vivo* and predicted by docking for AECS are consistent with those reported for each BC, identified by HPLC-QTOF-MS analysis, such as ellagic acid, which has been proposed to upregulate expression and activity of antioxidant enzymes (SOD, glutathione reductase, GPx, catalase) in murine models^(59)^. Another important BC is apigenin, which at low doses confers hepatoprotection by reducing lipid peroxidation and protein damage (0–40 mg/kg)^(60)^, whereas very high apigenin doses can paradoxically decrease antioxidant enzyme levels and induce hepatic OS (468–936 mg/kg)^(61)^. These dose-dependent effects underscore the importance of the extract matrix and administered dose in determining net biological outcomes. Thus, the phytochemical profile, *in vitro* antioxidant assays, *in vivo* pharmacological evaluation and *in silico* molecular docking analyses all converge to support the possible mechanism of the hypolipidaemic and antioxidant effects of AECS. The predominance of phenolic acids and flavonoids identified by HPLC-QTOF-MS provides a biochemical basis for the potent radical scavenging and metal reduction activities previously observed *in vitro*, which are reflected *in vivo* in the attenuation of hepatic oxidative damage to lipids and proteins and the normalisation of endogenous antioxidant defences (SOD and GPx) in the tyloxapol-induced hyperlipidaemia model. In parallel, molecular docking predicts that glycosylated flavonoids, particularly apigenin-7-O-glucuronide, as well as myricetin derivatives, interact favourably with key regulators of lipid metabolism, such as HMG-CoA reductase, PPAR*α*/*δ*/*γ* and LPL. This suggests a possible mechanistic link between the antioxidant capacity of the extract and the observed reduction in plasma TAG.

### Conclusions

Caco seeds could be considered a food with antioxidant and hypolipidaemic effects through their regulation of blood TAG levels, counteraction of adjacent oxidative stress and modulation of the antioxidant defense response. The antioxidant activity presented by the extracts is directly proportional to their phenol content. Molecular docking research has elucidated that apigenin-7-O–glucuronide presents a potent interaction with proteins related to lipid metabolism, including HMG-CoA reductase, PPAR*α*, PPAR*γ*, PPAR*δ* and LPL. The *in vivo* model demonstrated that aqueous extracts of Caco seeds have antioxidant activity. This effect could be beneficial in the body, for example, as a hepatoprotective, since it has been shown that its administration reduces oxidative stress in the liver, in addition to being able to control the lipid profile and the activity of antioxidant enzymes. In future research, it is recommended to conduct chronic preclinical and clinical studies, focused on determining the effectiveness and safety of Caco seed extract as an alternative treatment for diseases related to lipid metabolism.

## Table 1 Long description

A table comparing the concentrations of different bioactive compounds identified in the aqueous extract of Caco seed. The table has three rows and three columns. The columns are labeled 'Concentration', 'Mean', and 'SD'. The rows are labeled 'Saponin (µg DGE/g sample)', 'PC (µg PAE/g sample)', and 'Trypsin inhibitors (TIU)'. Row 1: Saponin, Mean 4730, SD 190. Row 2: PC, Mean 1.0, SD 0.05. Row 3: Trypsin inhibitors, Mean 6.14, SD 0.08. The table presents the mean concentrations and standard deviations of saponins, phytates, and trypsin inhibitors in the extract.

Navigate back to Table 1.

## Table 2 Long description

A table with 25 rows and 8 columns detailing the concentrations of phytochemical compounds in the aqueous extract of Caco seed. The columns are labeled as Number, rT (min), Theoretical m/z, Measured m/z, Error (amu), Signal intensity (cps), Formula, and Compounds. The table is divided into Positive mode and Negative mode sections. Each row lists specific data for each compound, including retention time, theoretical and measured mass-to-charge ratios, error in atomic mass units, signal intensity in counts per second, molecular formula, and compound name. The Positive mode section includes compounds like 3,4-O-dimethylgallic acid, (-)-Epigallocatechin, Apigenin 7-O-glucuronide, and 5,6,7,3,4 Pentahydroxyisoflavone. The Negative mode section includes compounds like Kaempferol 3-O-glucosylrhamnosyl-galactoside, Ellagic acid, p-hydroxybenzoic acid, and 4-Hydroxybenzoic acid 4-O-glucoside. The table provides detailed information on the identified compounds based on retention time, accuracy, and peak signal intensity.

Navigate back to Table 2.

## Table 3 Long description

A table comparing serum TAG levels and oxidative stress biomarkers in mice. The table has five columns: Groups, TAG (mmol/dl serum), LPO (mmol MDA/g tissue), POx (mmol CO•/g tissue), SOD (IU/g tissue), and GPx (IU/g tissue). Each column has Mean and SE sub-columns. The table has four rows labeled I, II, III (150), and IV (600). Row 1: Group I, TAG Mean 1.27, TAG SE 0.04, LPO Mean 39.52, LPO SE 2.6, POx Mean 253.12, POx SE 15.9, SOD Mean 688, SOD SE 53.9, GPx Mean 263.05, GPx SE 45.9. Row 2: Group II, TAG Mean 4.34, TAG SE 0.40, LPO Mean 45.35, LPO SE 1.1, POx Mean 336.55, POx SE 8.4, SOD Mean 986, SOD SE 42.3, GPx Mean 532.30, GPx SE 68.1. Row 3: Group III (150), TAG Mean 4.21, TAG SE 0.57, LPO Mean 42.55, LPO SE 2.5, POx Mean 316.55, POx SE 18.5, SOD Mean 701, SOD SE 76.5, GPx Mean 324.24, GPx SE 38.2. Row 4: Group IV (600), TAG Mean 1.56, TAG SE 0.18, LPO Mean 29.78, LPO SE 2.0, POx Mean 306.08, POx SE 19.3, SOD Mean 645, SOD SE 55.7, GPx Mean 686.15, GPx SE 87.2.

Navigate back to Table 3.

## Table 4 Long description

A table comparing binding affinities of various compounds with proteins related to lipid metabolism. The table has 25 rows and 6 columns. The columns are labeled Compound, HMG-CoA reductase, PPARα, PPARγ, PPARδ, and LPL. Each row lists a different compound and its binding affinity values for each protein. Row 1: 3,4-O-Dimethyl gallic acid, -5.9, -6.3, -6.2, -6.1, -5.7. Row 2: 4-Hydroxybenzoic acid 4-O-glucoside, -7.4, -7.7, -7.3, -8.0, -6.3. Row 3: 5,6,7,3,4-Pentahydroxy isoflavone, -8.2, -8.5, -8.1, -8.6, -7.8. Row 4: Apigenin-7-O-glucuronide, -9.3, -9.5, -9.2, -10.0, -8.4. Row 5: Dihydroferulic acid 4-O-glucuronide, -7.6, -8.0, -7.8, -8.4, -6.7. Row 6: Ellagic acid, -9.2, -7.8, -8.9, -8.3, -7.7. Row 7: Epicatechin, -8.5, -7.8, -7.9, -8.5, -7.8. Row 8: (-)-Epigallocatechin, -8.3, -7.5, -7.6, -8.4, -7.2. Row 9: Ferulic acid 4-O-glucuronide, -7.9, -8.4, -7.7, -8.3, -7.0. Row 10: Gallic acid, -6.4, -5.9, -5.9, -5.8, -6.0. Row 11: Gallic acid 4-O-glucoside, -7.5, -7.8, -7.1, -7.9, -6.2. Row 12: Kaempferol 3-O-glucosylrhamnosyl galactoside 1, -9.0, -6.2, -6.4, -7.8, -7.9. Row 13: Kaempferol-3,7-O-diglucoside 1, -8.3, -8.3, -8.4, -9.1, -8.0. Row 14: Myricetin, -8.7, -7.9, -8.2, -8.1, -7.7. Row 15: Myricetin 3-O-arabinoside, -7.4, -7.8, -7.6, -8.3, -6.9. Row 16: Myricetin 3-O-glucoside, -8.2, -8.0, -7.8, -8.3, -7.1. Row 17: Myricetin 3-O-glucuronide, -8.5, -8.4, -8.0, -8.5, -7.2. Row 18: Myricetin 3-O-rhamnoside, -8.4, -6.8, -7.6, -8.1, -8.1. Row 19: Myricetin 3-O-rutinoside, -9.0, -9.2, -6.9, -10.2, -7.6. Row 20: N-p-Coumaroyltyrosine, -8.0, -8.1, -8.4, -8.6, -7.7. Row 21: P-Coumaric acid, -6.5, -6.6, -6.5, -6.0, -6.4. Row 22: P-Coumaric acid4-O-glucoside, -7.4, -7.6, -7.5, -8.0, -6.9. Row 23: P-hydroxybenzoic acid, -5.9, -5.9, -5.8, -5.4, -5.6. Row 24: Protocatechuic acid, -6.4, -6.2, -6.0, -5.8, -6.0. Row 25: Quercetin, -8.7, -7.7, -8.4, -8.5, -7.6. Row 26: Control, -7.5, -7.0, -9.4, -9.7, -10.8.

Navigate back to Table 4.

## Table 5 Long description

The table presents data on the binding energies and interacting residues of various bioactive compounds with different receptors. It is organized into several sections based on the receptors: Compound/1HW8, Compound/PPARα, Compound/PPARγ, Compound/PPARδ, and Compound/lipoprotein lipase. Each section lists different compounds along with their binding energies and the specific residues they interact with. The table has multiple rows and columns, with column headers including Compound/1HW8, Energy, and Residues. Each row provides specific data for a compound, detailing its binding energy and the residues it interacts with. For example, under Compound/1HW8, Apigenin 7-O-glucuronide has a binding energy of -9.3 and interacts with residues such as Ala104, Cys105, Thr137, and others. Similar detailed information is provided for other compounds and receptors. The table highlights the interactions and binding affinities of these compounds, showing how they bind to specific residues of the target proteins.

Navigate back to Table 5.
